# Mosaic Trisomy 14 with Severe Short Stature: A Case Report

**DOI:** 10.3390/genes17091123

**Published:** 2026-09-15

**Authors:** Chunyan Yin, Juan Ye, Ling Hou, Xiaoping Luo

**Affiliations:** 1 Department of Pediatrics, Hubei Provincial Key Laboratory of Pediatric Genetic Metabolic and Endocrine Rare Diseases, Hubei Provincial Clinical Research Center for Children’s Growth and Development and Metabolic Diseases, State Key Laboratory for Diagnosis and Treatment of Severe Zoonotic Infectious Disease, Tongji Hospital, Tongji Medical College, Huazhong University of Science and Technology, Wuhan 430030, China; 2 Department of Pediatrics, Second Affiliated Hospital of Xi’an Jiaotong University, Xi’an 710049, China

**Keywords:** chromosomal microarray analysis, mosaic trisomy 14, recombinant human growth hormone, severe short stature, small for gestational age, 14q32.2 allele dosage

## Abstract

Mosaic trisomy 14 is a rare chromosomal anomaly with a broad phenotypic spectrum. We report a 10-year-3-month-old girl with severe short stature, developmental delay, and repaired patent ductus arteriosus. An incompletely documented combined insulin–clonidine stimulation test yielded a peak GH concentration of 6.85 ng/mL and was not considered sufficient to establish growth hormone deficiency. Karyotyping of 100 peripheral-blood metaphases showed 47,XX,+14[6]/46,XX[94]. Initial copy-number sequencing detected a 34.37 Mb mosaic 14q gain; repeat SNP-based chromosomal microarray analysis demonstrated an approximately 86.83 Mb 14q11.2-q32.33 mosaic gain at an array-estimated fraction of approximately 50%, compatible with the cytogenetic diagnosis. Targeted 14q32.2 analysis showed increased total and methylated-allele dosage, with methylated fractions of 48.6–62.3%, interpreted as dosage imbalance within the broader 14q gain rather than an independent epimutation. Four STR loci showed biparental inheritance. Quantitative peak-height and peak-area analysis at three informative, bias-correctable STR loci demonstrated excess paternal-allele dosage, providing independent support for, but not definitive proof of, paternal origin of the additional chromosome 14. rhGH was prescribed for SGA with persistent short stature at 0.22 mg/kg/week. At three months, height was 124.7 cm (+1.9 cm); no adverse events were reported. These observations do not establish treatment efficacy or safety.

## 1. Introduction

Trisomy 14 is an exceptionally rare autosomal aneuploidy. Complete trisomy 14 is generally incompatible with live birth and usually results in early embryonic loss or spontaneous miscarriage, whereas mosaic trisomy 14 represents a viable form of this chromosomal abnormality and is associated with a broad range of clinical phenotypes [1,2]. Since the initial description by Rethoré et al. in 1975, fewer than 100 live-born cases have been systematically reported in the literature [3]. The phenotype commonly involves multiple organ systems, including fetal growth restriction and postnatal growth failure, intellectual and motor developmental delay, congenital heart defects, dysmorphic facial features, pigmentary skin abnormalities, and skeletal anomalies [4].

A major diagnostic consideration in any child carrying a supernumerary or rearranged chromosome 14 is the potential coexistence of imprinting abnormalities involving the 14q32.2 region, which may arise through a trisomy-rescue mechanism and produce either maternal uniparental disomy of chromosome 14 [UPD(14)mat]/Temple syndrome (loss of methylation at the IG-DMR/MEG3-DMR) or paternal uniparental disomy of chromosome 14 [UPD(14)pat]/Kagami–Ogata syndrome (gain of methylation at the same DMRs) [5,6,7,8]. Both imprinting disorders share several features with mosaic trisomy 14, including growth failure, hypotonia, developmental delay, small hands and feet, and pubertal or body-composition abnormalities, and the distinction cannot be made clinically at a single time point [5,6,7,8,9,10,11]. Because of the rarity of mosaic trisomy 14 and its phenotypic overlap with chromosome 14 imprinting disorders, diagnosis is often delayed or missed. We report a girl with mosaic trisomy 14, severe persistent short stature, and a subnormal GH response on an incompletely documented stimulation test, further characterized by initial copy-number variation sequencing (CNV-seq), repeat high-density SNP-based chromosomal microarray analysis, and targeted methylation-specific copy-number and STR analyses. The case is reported in accordance with the CARE guidelines [12], and the diagnostic and management implications are discussed in the context of the available literature.

## 2. Case Presentation

### 2.1. General Information

The patient, a 10-year-and-3-month-old girl, was admitted to the Department of Pediatric Endocrinology, Genetics, Metabolism and Rare Diseases, Tongji Hospital, Tongji Medical College, Huazhong University of Science and Technology, on 26 January 2026, because of dysmorphic facial features, growth failure, and a history of congenital heart disease. She was the second child of non-consanguineous parents and was delivered vaginally at 37 weeks of gestation. Birth weight was 2.4 kg, corresponding to approximately the 14th percentile (−1.1 SDS) for gestational age and sex. Birth length was 43 cm, corresponding to approximately −2.4 SDS, consistent with short birth length for gestational age according to INTERGROWTH-21st newborn standards [13]. There was no postnatal hypoxia or asphyxia. The pregnancy had been complicated by fetal growth restriction (FGR), oligohydramnios, and decreased fetal movements. Developmental milestones were delayed: head control and tooth eruption at 5 months, independent sitting at 8 months, first words at 18 months, and independent walking at 20 months. At 1 year of age, a continuous murmur was detected in the left second intercostal space, and echocardiography subsequently identified patent ductus arteriosus (PDA), which was surgically treated at 1.5 years of age. The father’s height was 178 cm, the mother’s height was 168 cm, and the mid-parental target height was 166.5 ± 4 cm. There was no family history of genetic disease or congenital malformation. This study was approved by the Ethics Committee of Tongji Hospital, Tongji Medical College, Huazhong University of Science and Technology (Approval No. TJ-C20260112, Date of Approval: 12 January 2026). Written informed consent was obtained from the patient’s legal guardians for participation and for publication of clinical and genetic information. The study was conducted in accordance with the Declaration of Helsinki.

### 2.2. Physical Examination

**Figure 1:**
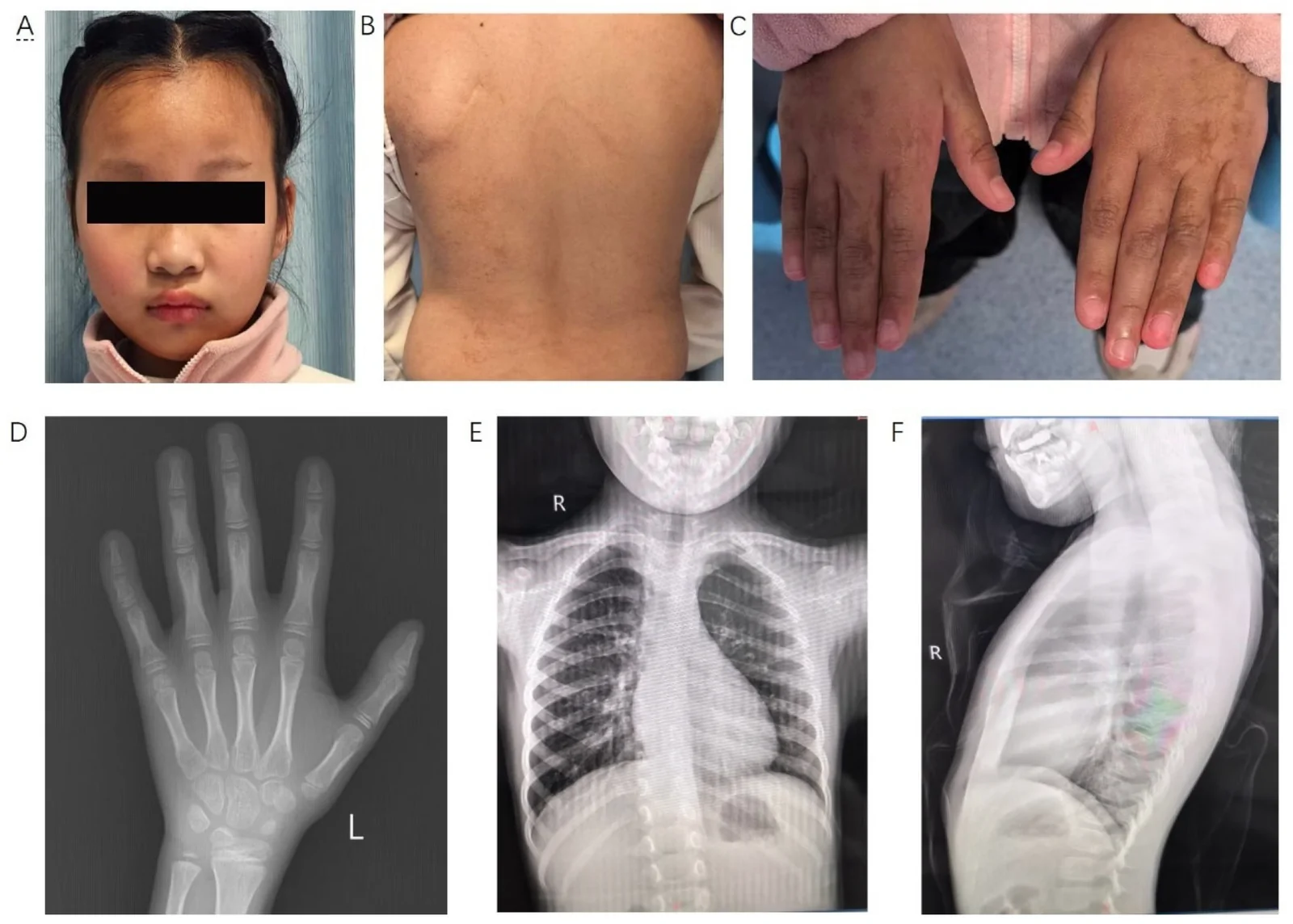
Clinical features of the 10-year-and-3-month-old female patient with mosaic trisomy 14 and severe short stature. (**A**) Facial features including hypertelorism, a low nasal bridge, short philtrum, upslanted palpebral fissures, and facial asymmetry; (**B**) scoliosis, back hyperpigmentation, and postoperative scar from PDA surgery; (**C**) small hands and pigmentary abnormalities on the hands; (**D**) left hand X-ray showing delayed bone age (6 years 8 months at chronological age 10 years 3 months); (**E**,**F**) whole-spine X-ray anteroposterior and lateral views showing right-convex thoracolumbar scoliosis (Cobb angle 13°) and thoracic kyphosis (30°).

Physical examination showed a height of 122.8 cm (<3rd percentile, −2.75 SDS), weight of 24.0 kg (3rd–10th percentile), body mass index of 15.92 kg/m^2^ (15th–50th percentile), sitting height of 67 cm, arm span of 113 cm, and head circumference of 50 cm (approximately −1.6 SDS for age and sex). Validated age- and sex-specific SDS reference datasets were available in the clinical record for height and head circumference; weight and body mass index are reported as percentiles, and sitting height and arm span are reported as measured values because appropriate reference datasets for these parameters were not available in the clinical record. Dysmorphic facial features included hypertelorism, a low nasal bridge, short philtrum, upslanted palpebral fissures, and facial asymmetry (Figure 1A). The patient also had a short neck, small hands and feet, bilateral genu valgum with the left lower limb approximately 2 cm shorter than the right, and a leg-length discrepancy of approximately 2 cm. Scattered hyperpigmented lesions were present on the trunk, limbs, buttocks, and hands (Figure 1B,C). Pubertal development was prepubertal, with Tanner stage 1 breasts, normal areolar pigmentation, and no pubic or axillary hair. Body composition and weight gain velocity were within the prepubertal reference range, and there was no clinical evidence of truncal obesity.

### 2.3. Auxiliary Laboratory and Imaging Findings

Laboratory testing showed normal complete blood count, blood glucose, liver and kidney function, and thyroid function. Serum 25-hydroxyvitamin D [25(OH)D], measured by electrochemiluminescence immunoassay (Roche Cobas e801; laboratory reference interval 30–100 µg/L), was decreased at 21.47 µg/L. Insulin-like growth factor-1 (IGF-1), measured by chemiluminescent immunoassay (Siemens Atellica IM; assay coefficient of variation < 8%), was 87 µg/L, with an age- and sex-adjusted SDS of −1.97 calculated using the Siemens Atellica IGF-1 analyzer normative reference. Sex hormone results were consistent with a prepubertal state: luteinizing hormone (LH) < 0.3 mIU/mL, follicle-stimulating hormone (FSH) 4.34 mIU/mL, and estradiol < 5 pg/mL. IGFBP-3 was not measured in this patient.

A combined insulin–clonidine GH stimulation session was performed during the endocrine evaluation. Re-review of contemporaneous medication orders showed insulin prescribed at 0.075 U/kg and clonidine 0.096 mg orally (approximately 4 µg/kg for the patient’s body weight). The archived pituitary-function laboratory report documented GH concentrations of 0.75 ng/mL at 0 min, 1.99 ng/mL at 30 min, 5.65 ng/mL at 60 min, and 6.85 ng/mL at 90 min, with 6.85 ng/mL as the highest recorded value (Table 1). The 15 and 120 min fields contained no GH values. Serial glucose concentrations, exact administration times relative to blood sampling, and the original GH assay platform/calibration standard were not preserved in the retrieved records. Therefore, adequate insulin-induced hypoglycemia cannot be verified, the combined session cannot be interpreted as two independent provocative tests, and the result is not sufficient to establish growth hormone deficiency. The peak of 6.85 ng/mL is reported descriptively as a subnormal stimulated GH response under some historical cutoffs, recognizing that GH thresholds are assay- and protocol-dependent [14]. The patient’s severe short stature, delayed bone age, clinical record of poor linear growth, and IGF-1 of 87 µg/L (−1.97 SDS) provide relevant auxological and biochemical context but are not independently diagnostic of GHD.

Pituitary and brain magnetic resonance imaging (MRI) showed no abnormalities of the hypothalamic–pituitary axis. Left hand radiography showed delayed bone age (Figure 1D), assessed by the Greulich–Pyle method as 6 years and 8 months at a chronological age of 10 years and 3 months (bone-age delay approximately 3 years and 7 months); the corresponding bone age at the 3-month follow-up was 6 years and 11 months. Whole-spine radiography showed a right-convex thoracolumbar scoliosis with a Cobb angle of 13° and a thoracic kyphosis of 30° (Figure 1E,F). Pelvic ultrasonography showed a normal uterine size and small follicles in both ovaries (largest follicle: left 0.4 cm, right 0.3 cm), consistent with prepubertal status. Thyroid ultrasonography, performed as part of the standard endocrine work-up for short stature, showed a small cystic lesion in the left lobe that was classified as TI-RADS 2 (benign) and required no further intervention. Echocardiography showed no residual shunt after PDA closure, normal left ventricular systolic function (ejection fraction 68%), and mild mitral and tricuspid regurgitation. Abdominal and adrenal ultrasonography showed no abnormalities. Electrocardiography showed sinus arrhythmia, sinus tachycardia, and left anterior fascicular block; outpatient cardiology review judged these findings to be clinically non-specific and recommended routine clinical follow-up without specific treatment. The Wechsler Intelligence Scale for Children, Fourth Edition (WISC-IV), administered by a licensed psychologist, showed a verbal comprehension index of 45, a perceptual reasoning index of 58, and a full-scale IQ of 50. Because adaptive functioning was not formally assessed with an adaptive behavior scale, intellectual disability is reported descriptively (full-scale IQ 50) and is not labeled by severity.

### 2.4. Karyotype, Copy-Number Sequencing, and Chromosomal Microarray Analysis

**Figure 2:**
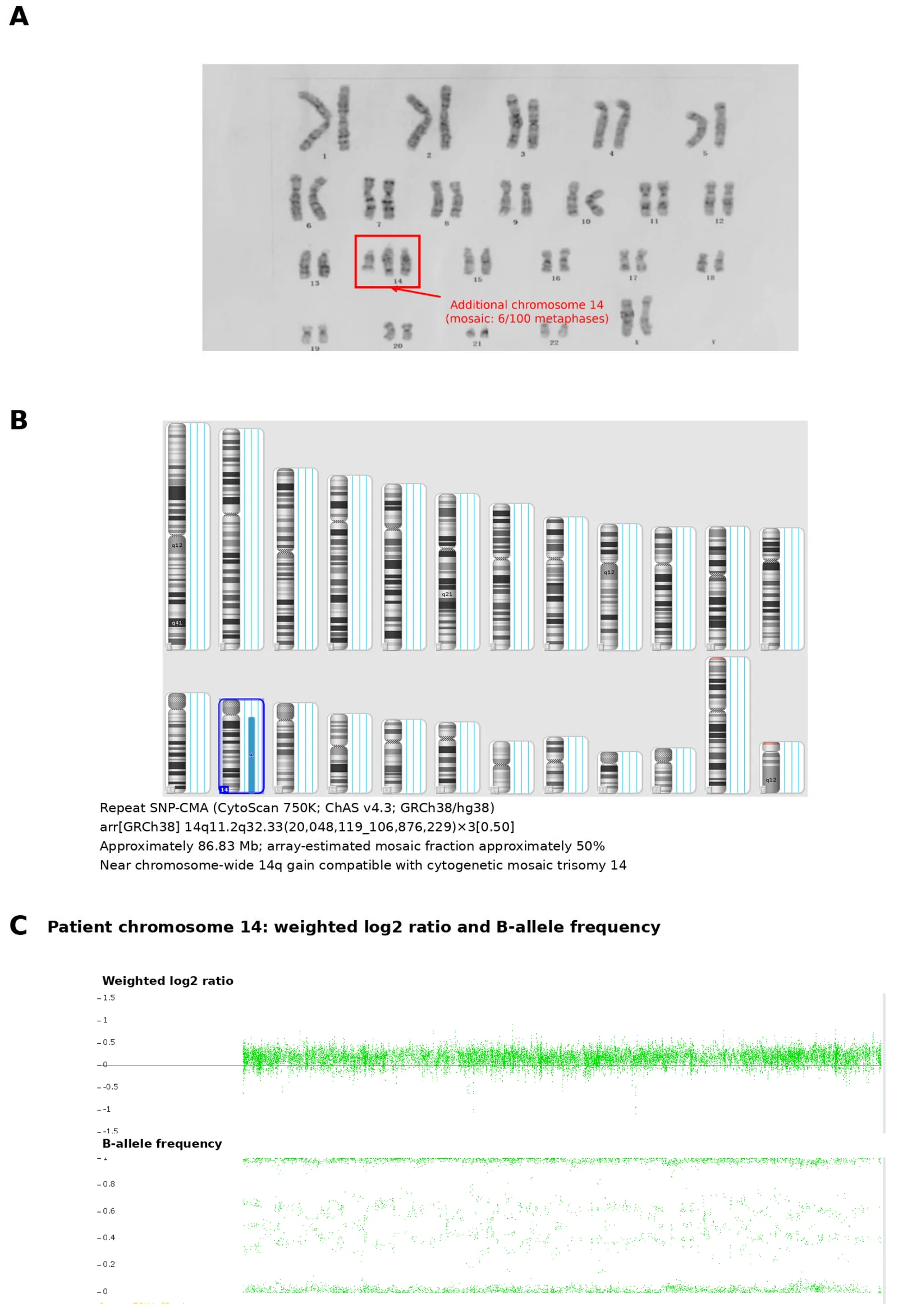
Cytogenetic and chromosomal microarray characterization of mosaic chromosome 14 gain. (**A**) Representative G-banded karyogram showing the 47,XX,+14 cell line identified in 6 of 100 cultured peripheral-blood metaphases; the red box and arrow mark the chromosome 14 group containing the additional chromosome. The laboratory-issued ISCN 2024 notation was 47,XX,+14[6]/46,XX[94]. (**B**) Repeat CytoScan 750K chromosomal microarray ideogram (ChAS v4.3; GRCh38/hg38) demonstrating a broad mosaic gain spanning 14q11.2-q32.33, reported by the laboratory under ISCN 2020 as arr[GRCh38] 14q11.2q32.33(20,048,119_106,876,229)×3[0.50] (approximately 86.83 Mb; array-estimated mosaic fraction approximately 50%). Because the repetitive short arm of chromosome 14 is not directly interrogated, this is described as a near chromosome-wide 14q gain compatible with trisomy 14. (**C**) Laboratory-issued chromosome 14 weighted log2-ratio and B-allele-frequency export from the patient. These plots support the broad 14q mosaic gain but are not used to assign parent of origin or to resolve the quantitative difference between cultured metaphases and DNA-based mosaic estimates. Parent-of-origin inference is based on the independent quantitative STR analysis shown in Supplementary Figure S1 and Table S1, not on the CMA plots alone.

GTG-banded karyotype analysis of 100 peripheral-blood metaphase cells (Beijing Haisite Medical Laboratory; MetaSystems imaging platform (Thermo Fisher Scientific, Waltham, MA, USA)g platform; banding resolution approximately 400–450 bands per haploid genome; phytohemagglutinin-stimulated 72 h lymphocyte culture) identified six cells with trisomy 14 and 94 cells with a normal female karyotype. The signed clinical cytogenetic report, issued under ISCN 2024, used the notation 47,XX,+14[6]/46,XX[94]; this laboratory-issued notation is reproduced verbatim (Figure 2A). Initial genome-wide copy-number variation analysis on peripheral-blood DNA was performed at Beijing MyGenostics Co., Ltd., Beijing, China using capture-based next-generation sequencing (CNV-seq; CapNGS; GRCh37/hg19). It identified a mosaic copy-number gain at 14q11.2-q22.3 (chr14:22,749,742-57,116,759), approximately 34.37 Mb, with a laboratory-estimated mosaic fraction of approximately 48% based on normalized sequencing depth. Because this assay did not genotype SNPs, B-allele-frequency data were unavailable. Repeat high-density SNP-based chromosomal microarray analysis was subsequently performed at Beijing Haisite Medical Laboratory using the Affymetrix CytoScan 750K platform (approximately 550,000 non-polymorphic copy-number probes and 200,000 genotype-able SNP probes), ChAS v4.3, and GRCh38/hg38. The signed array report, issued using ISCN 2020, stated arr[GRCh38] 14q11.2q32.33(20,048,119-106,876,229)×3[0.50], representing an approximately 86.83 Mb broad mosaic 14q gain with an array-estimated mosaic fraction of approximately 50%. Because the repetitive short arm of acrocentric chromosome 14 is not directly assessed by the array, this finding is described as a near chromosome-wide 14q gain compatible with the cytogenetic diagnosis of mosaic trisomy 14 rather than as independent proof of gain of every chromosome 14 component. The laboratory-issued chromosome 14 weighted log2-ratio and B-allele-frequency export is shown in Figure 2C. The similar estimates from the two DNA-based assays do not resolve the substantial quantitative discrepancy with the 6% abnormal cultured metaphases; no independent quantitative assay on uncultured cells was available. The parents’ microarray results showed no chromosome 14 copy-number abnormality. Parental G-banded karyotypes analyzed in 100 metaphases each were 46,XX[100] and 46,XY[100]; no visible balanced chromosome 14 rearrangement was detected at the reported G-banding resolution, although cryptic rearrangements below that resolution cannot be excluded. FISH, ddPCR, qPCR, buccal-cell testing, and fibroblast testing were not available.

### 2.5. Methylation-Specific Copy-Number Assay and Str Linkage Analysis

To address the differential diagnosis of chromosome 14 imprinting disorders, peripheral-blood samples from the patient and both parents were submitted to Cipher Gene (Beijing, China) for a clinical methylation-specific AccuCopy^®^ assay combined with multiplex fluorescent STR genotyping and copy-number analysis (reference genome GRCh38/hg38). The panel covered six genomic imprinting regions: 6q24.2 (PLAGL1/HYMAI), 7q32.2 (MEST), 11p15 (H19-DMR/IGF2 and Kv-DMR/CDKN1C/KCNQ1OT1), 14q32.2 (IG-DMR, MEG3-DMR, MEG8-DMR within the DLK1/DIO3 locus), 15q11.2-q13 (SNURF-SNRPN/UBE3A), and 20q13.32 (GNAS locus). The methylation-specific copy-number assay quantifies total copy number as well as methylated and unmethylated allele copy numbers at each differentially methylated region (DMR); the STR panel provides biparental inheritance information and detects segmental UPD.

**Figure 3:**
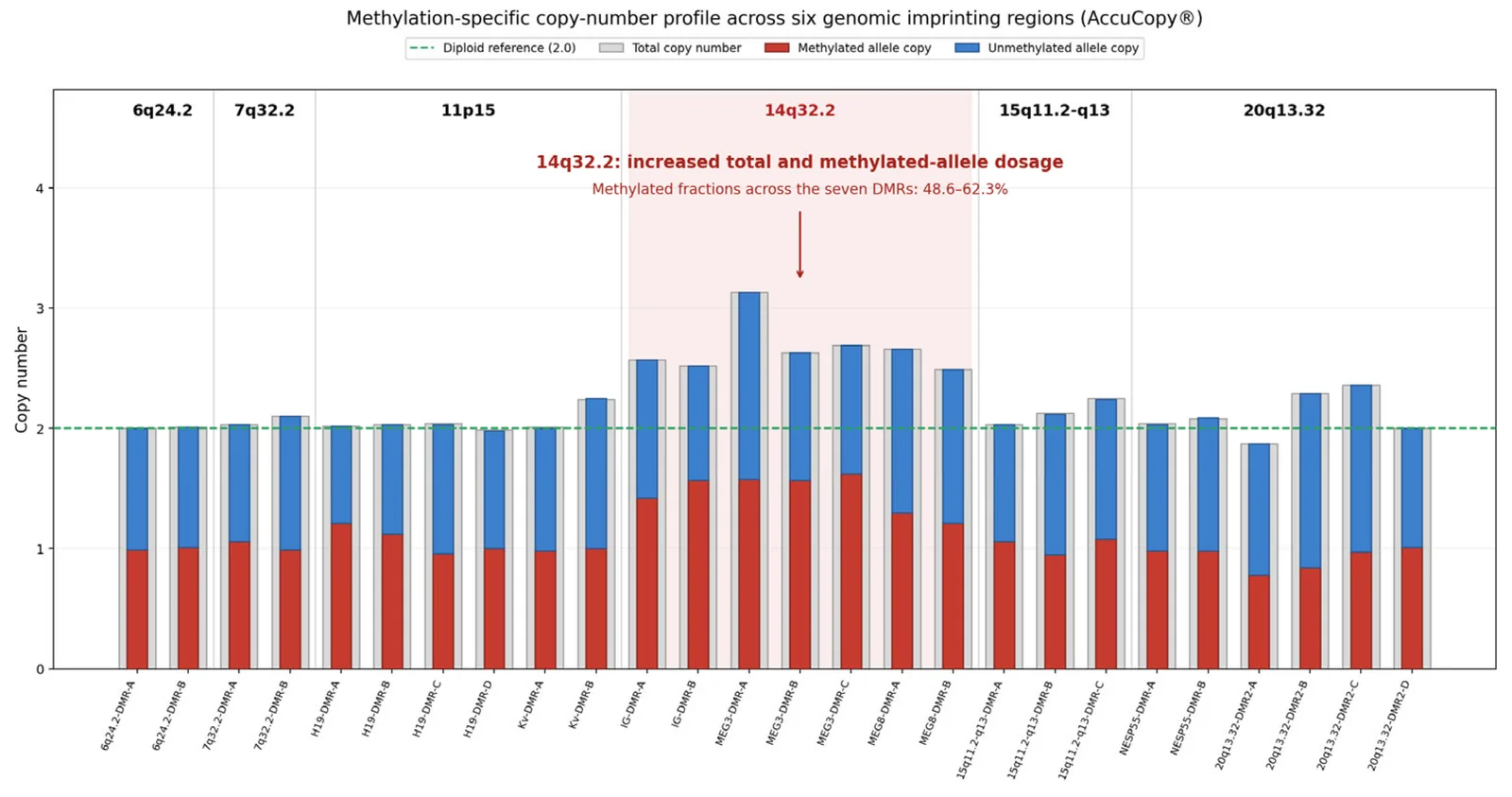
Methylation-specific copy-number profile across six genomic imprinting regions. The AccuCopy^®^ assay showed increased total and methylated-allele copy numbers at the 14q32.2 DMRs. Methylated fractions, calculated as methylated-allele copy number divided by total copy number, were 55.3%, 62.3%, 50.5%, 59.7%, 60.2%, 48.9%, and 48.6% across IG-DMR-A, IG-DMR-B, MEG3-DMR-A, MEG3-DMR-B, MEG3-DMR-C, MEG8-DMR-A, and MEG8-DMR-B, respectively. Because the laboratory report did not provide validated proportional methylation reference intervals and the 14q32.2 interval lies within the broader mosaic 14q gain, these findings are interpreted as increased methylated-allele dosage rather than an independently demonstrated epimutation. Other assayed imprinting regions showed approximately diploid total copy number and balanced absolute methylated/unmethylated copy-number profiles.

Methylation-specific copy-number analysis showed increased total copy number at the 14q32.2 DMRs (2.49–3.13), with methylated-allele copy numbers of 1.21–1.62 and unmethylated-allele copy numbers of 0.95–1.55 (Table 2; Figure 3). For each DMR, the methylated fraction was calculated as methylated-allele copy number divided by total copy number: IG-DMR-A 55.3%, IG-DMR-B 62.3%, MEG3-DMR-A 50.5%, MEG3-DMR-B 59.7%, MEG3-DMR-C 60.2%, MEG8-DMR-A 48.9%, and MEG8-DMR-B 48.6%. The signed clinical report flagged increased absolute methylated-copy dosage but did not provide validated proportional methylation reference intervals. Accordingly, the revised manuscript does not interpret these values as evidence of an independent epimutation. The targeted assay covered chr14:100,810,639-100,982,948 (GRCh38/hg38), an approximately 172.3 kb interval wholly contained within the broader 14q11.2-q32.33 mosaic gain. Without breakpoint-resolving evidence, this targeted result is treated as confirmation of increased copy number within the larger gain rather than as a structurally independent 172.3 kb duplication. STR analysis at four 14q32.2 loci (G14S19, G14S23, G14S26, G14S28) demonstrated alleles compatible with both parents in peripheral blood. These allele-size data provide no evidence of UPD(14) at the assayed loci but do not establish the dosage origin of the third chromosome 14 copy.

Several 14q32.2 DMRs showed methylated fractions close to 50% despite increased total copy number, while others were modestly above 50%. In the setting of mosaic trisomy 14, this pattern is compatible with increased dosage of a normally methylated allele in the trisomic cell population and does not require a primary gain-of-methylation epimutation. The present blood-based data therefore do not support the loss-of-methylation pattern characteristic of UPD(14)mat/Temple syndrome [6,8,11], and the four assayed STR loci show biparental inheritance; however, tissue-restricted UPD(14) or imprinting mosaicism outside peripheral blood cannot be excluded. The 14q32.2 findings are therefore described as increased total and methylated-allele dosage within the broader mosaic 14q gain rather than as a separately demonstrated epimutation.

Archived STR fragment-analysis electropherograms and quantitative peak metrics were reanalyzed to assess parental allele dosage. At G14S19, G14S23, and G14S26, an identical heterozygous allele pair in one parent allowed correction for allele-specific PCR amplification bias. The normalized paternal-to-maternal ratios were approximately 1.63, 1.36, and 1.26 by peak height and 1.62, 1.39, and 1.28 by peak area, respectively; the corresponding mean normalized ratios were 1.42 and 1.43. At G14S28, the paternal-specific 462 bp allele and maternal-specific 456 bp allele were distinguishable, but no matched parental heterozygous allele pair was available for amplification-bias correction, so this marker was not included in the quantitative mean. The three bias-correctable markers therefore show excess paternal-allele dosage, consistent with the approximately 1.5 paternal:maternal dosage expected in a theoretical 50% trisomic mixture containing one additional paternal chromosome 14 copy. These quantitative STR data provide independent support for, but do not definitively prove, paternal origin of the additional chromosome 14 (Supplementary Figure S1 and Table S1).

The methylation-specific copy-number assay was performed by Cipher Gene (Beijing, China) using AccuCopy^®^ technology. The methylated fraction was calculated as methylated-allele copy number divided by total copy number ×100. The signed laboratory report provided absolute total, methylated, and unmethylated copy numbers but did not provide validated proportional methylation reference intervals. Therefore, the calculated fractions are reported descriptively and are not interpreted as an independent epimutation. The targeted 172.3 kb interval lies within the broader 14q11.2-q32.33 mosaic gain and is not interpreted as a structurally independent duplication. STR analysis at four 14q32.2 loci demonstrated biparental alleles. Reanalysis of archived fragment-analysis peak metrics showed normalized paternal-to-maternal ratios of 1.63/1.62 (peak height/area) at G14S19, 1.36/1.39 at G14S23, and 1.26/1.28 at G14S26; mean normalized ratios were 1.42 by peak height and 1.43 by peak area. These values support excess paternal-allele dosage but are not considered definitive proof of parent of origin. G14S28 distinguished paternal-specific 462 bp and maternal-specific 456 bp alleles but was not included in the normalized mean because no matched parental heterozygous pair was available for amplification-bias correction (Supplementary Figure S1 and Table S1).

### 2.6. Treatment and Three-Month Follow-Up

The formal indication for recombinant human growth hormone (rhGH) recorded in the medical record was small for gestational age (SGA) with persistent short stature, rather than a definitive diagnosis of GHD. Birth length was −2.4 SDS, meeting the international consensus definition of SGA by length (<−2 SDS), whereas birth weight was −1.1 SDS; at 10 years 3 months, height remained −2.75 SDS. Serial early-childhood height measurements were insufficient to reconstruct the exact timing and magnitude of spontaneous catch-up growth. rhGH (GeneScience Pharmaceuticals; lyophilized powder for subcutaneous injection) was started on 27 January 2026, at the charted dose of 0.22 mg/kg/week (approximately 0.031 mg/kg/day), administered subcutaneously. This dose is close to the international consensus starting dose of 0.033 mg/kg/day for persistent short stature after SGA birth [15]. The medical record did not document a separate local eligibility statement specific to mosaic trisomy 14; therefore, the manuscript reports the individualized treatment decision without generalizing eligibility under local guidance. Because serum 25(OH)D was low (21.47 µg/L) and dietary calcium intake was below the recommended intake for age, cholecalciferol (vitamin D3, 800 IU/day) and calcium carbonate (500 mg elemental calcium/day) were supplemented. Dual-energy X-ray absorptiometry was not performed. Because of scoliosis and leg-length discrepancy, the patient was fitted with a custom thoracolumbar orthosis and corrective shoes, and motor and speech rehabilitation were provided.

At the three-month visit, height was 124.7 cm compared with 122.8 cm at baseline, an observed interval increase of 1.9 cm; weight was 25.1 kg compared with 24.0 kg (Table 3). Serum IGF-1 was 145 µg/L (−0.55 SDS) compared with 87 µg/L (−1.97 SDS) at baseline, and 25(OH)D was 32 µg/L compared with 21.47 µg/L. The Cobb angle was 13° at both examinations. Fasting glucose was 4.83 mmol/L versus 4.56 mmol/L, and HbA1c was 5.2% at both time points; liver, kidney, and thyroid function remained within the reference range. Bone age was read as 6 years 11 months at the three-month visit versus 6 years 8 months at baseline, but this small interval was not interpreted as treatment-related because it is within the precision limitations of the Greulich–Pyle method. No adverse events were reported during the three months of observation, and family-recorded adherence exceeded 95%. The rhGH dose was not adjusted during this interval. Because a reliable pretreatment growth velocity could not be independently recalculated and no untreated comparator was available, these observations are reported descriptively and do not establish treatment efficacy or safety.

Formal rhGH indication: SGA with persistent short stature. Birth length was −2.4 SDS, meeting SGA by length; birth weight was −1.1 SDS. The charted rhGH dose was 0.22 mg/kg/week (approximately 0.031 mg/kg/day), administered subcutaneously. The available early-childhood measurements were insufficient to reconstruct the exact timing of failed spontaneous catch-up growth.

## 3. Discussion

### 3.1. Clinical Phenotype of Mosaic Trisomy 14

Mosaic trisomy 14 is a rare chromosomal disorder with a variable clinical spectrum. Clinical severity is influenced by the proportion of trisomic cells and their tissue distribution. Published cases show recurring features involving growth, neurodevelopment, the cardiovascular system, the skeleton, the skin, the endocrine system, and the face [4,16,17,18]. The present patient exhibited many typical manifestations, including fetal growth restriction and postnatal growth failure, congenital heart disease in the form of surgically repaired PDA, mild scoliosis, pigmentary skin abnormalities, small hands and feet, limb asymmetry, and delayed motor and cognitive development (Table 4). These observations are consistent with the recognized phenotypic spectrum of mosaic trisomy 14, but several features warrant closer consideration in the context of chromosome 14 imprinting.

### 3.2. Differential Diagnosis from Upd(14)mat and Upd(14)pat

Mosaic trisomy 14 may coexist with UPD(14), including UPD(14)mat/Temple syndrome, after trisomy rescue [5,6,7,8,9,19]. The two chromosome 14 imprinting disorders share several features with mosaic trisomy 14, including pre- and postnatal growth failure, hypotonia, motor and cognitive delay, small hands and feet, and pubertal or body-composition abnormalities [5,6,7,8,9,10,11]. In the present case, the 14q32.2 targeted assay showed increased total and methylated-allele dosage, but proportional methylation across the seven DMRs ranged from 48.6% to 62.3% and several loci were approximately balanced. These findings do not establish an independent epimutation. STR analysis at G14S19, G14S23, G14S26, and G14S28 demonstrated biparental alleles in peripheral blood and provided no evidence of UPD(14) at those loci. Reanalysis of quantitative electropherogram peak height and peak area at three informative, bias-correctable markers demonstrated excess paternal-allele dosage, supporting a paternal origin of the additional chromosome 14 without establishing it definitively (Supplementary Figure S1 and Table S1). Because all imprinting and STR analyses were blood-based, tissue-restricted UPD(14) or imprinting mosaicism outside blood cannot be completely excluded, a limitation relevant to trisomy-rescue states [19]. The differential diagnosis for syndromic growth failure remains broad and includes other chromosomal and imprinting disorders [20,21]. In this case, karyotyping together with repeat SNP-based CMA supports the chromosomal diagnosis, whereas the 14q32.2 assay is interpreted as allele-dosage information within the broader mosaic 14q gain rather than as a separate imprinting disorder. A cautious comparison with Temple and Kagami–Ogata syndromes is summarized in Table 5.

### 3.3. Subnormal Gh Response and Rhgh Treatment for Persistent Short Stature After Sga Birth

The available GH stimulation study does not reliably establish GHD. The archived report documented a highest measured GH concentration of 6.85 ng/mL in an incompletely documented combined insulin–clonidine session, without serial glucose values, exact administration times, the 15 and 120 min GH samples, or preserved assay/calibration details. Provocative GH testing is assay- and protocol-dependent and should be interpreted with auxology and other clinical data rather than as a stand-alone diagnostic threshold [14]. In this patient, IGF-1 was borderline low (−1.97 SDS), pituitary MRI was normal, and a complete set of dated pretreatment height measurements was unavailable; additionally, the chromosomal disorder and SGA by length provide alternative explanations for severe short stature. Accordingly, the manuscript no longer diagnoses partial or complete GHD and reports the stimulation result only as a subnormal response in an incompletely documented test. The formal rhGH indication was SGA with persistent short stature, and the charted dose was 0.22 mg/kg/week, close to the consensus starting dose for this indication [15]. At three months, height had increased by 1.9 cm and IGF-1 SDS had changed from −1.97 to −0.55, but the absence of a reliably calculated pretreatment velocity or untreated trajectory precludes causal attribution. The Cobb angle was 13° at both examinations; three months is insufficient to assess scoliosis risk, and longer monitoring is warranted. Although rhGH has been reported in Temple syndrome [22], those observations concern a different molecular diagnosis and are not used to interpret the present GH stimulation study. Recent cohort data have examined scoliosis during rhGH treatment in pediatric populations, but these observational data do not establish causality [23].

### 3.4. Diagnostic Pathway and Integration of Cytogenetic, Molecular, and Imprinting Data

Conventional G-banded karyotyping identified trisomy 14 in 6 of 100 cultured peripheral-blood metaphases. Initial CNV-seq and repeat SNP-based CMA on peripheral-blood DNA yielded similar DNA-based mosaic estimates of approximately 48% and 50%, respectively, and the repeat CMA demonstrated a broad 14q11.2-q32.33 gain compatible with the cytogenetic diagnosis. However, the large quantitative difference between 6% abnormal cultured metaphases and approximately 50% in uncultured leukocyte DNA remains unresolved. Differential survival or proliferation during phytohemagglutinin-stimulated culture, differences in cell populations, and assay-specific estimation are possible explanations but remain hypotheses in this patient. No independent quantitative confirmation using interphase FISH, ddPCR, qPCR, buccal cells, or skin fibroblasts was available. The high-resolution chromosome 14 weighted log2-ratio and BAF export is provided in Figure 2C, but these data do not by themselves resolve the culture-versus-DNA mosaic-fraction discrepancy. The repeat array demonstrates a near chromosome-wide 14q gain; because the repetitive acrocentric short arm is not directly interrogated, the array result is described as compatible with trisomy 14 rather than as independent proof of gain of every chromosome 14 component. The targeted 14q32.2 assay lies entirely within this broader gain and is interpreted as increased allele dosage within the same mosaic chromosome 14 material, not as an independent structural duplication.

Parental G-banded karyotyping did not detect a balanced chromosome 14 rearrangement at the reported 400–450-band resolution (mother 46,XX[100]; father 46,XY[100]); cryptic rearrangements below that resolution cannot be excluded. Because mosaicism may differ across tissues, the mosaic fraction in peripheral blood may not represent that in other tissues; buccal-cell or skin-fibroblast testing could provide additional quantitative information if clinically indicated [20,21]. The prenatal history of fetal growth restriction and oligohydramnios underscores the value of prenatal evaluation when fetal growth abnormalities are present. A recent prenatal report of high-level mosaic trisomy 14 likewise described congenital heart defects and fetal growth restriction [24]. Genome-wide non-invasive prenatal testing may identify rare autosomal trisomies, but positive results require confirmation by invasive diagnostic testing [25]. Genetic counseling should emphasize the rarity and variable prognosis of mosaic trisomy 14, possible tissue-specific mosaicism, and the limits of recurrence-risk inference from the present testing.

The 14q32.2 data are most appropriately interpreted as allele-dosage measurements within the broader mosaic 14q gain. Across the seven assayed DMRs, methylated fractions ranged from 48.6% to 62.3%; several loci were close to 50% despite increased total copy number, making increased dosage of a normally methylated allele a sufficient explanation without invoking an independent epimutation. The clinical laboratory report did not provide validated proportional methylation reference intervals, so no independent methylation-index abnormality is claimed. STR allele sizes at four loci demonstrated biparental inheritance in peripheral blood and provided no evidence of UPD(14) at the assayed loci. Quantitative reanalysis of archived electropherograms showed normalized paternal-to-maternal peak-area ratios of approximately 1.62, 1.39, and 1.28 at G14S19, G14S23, and G14S26, respectively, with concordant peak-height ratios of approximately 1.63, 1.36, and 1.26; the mean normalized ratios were 1.43 by peak area and 1.42 by peak height. These values are consistent with excess paternal-allele dosage in the setting of approximately 50% trisomy-14 mosaicism and provide independent support for, but not definitive proof of, paternal origin of the additional chromosome 14. G14S28 independently distinguished a paternal-specific 462 bp allele from a maternal-specific 456 bp allele but was not used for normalized dosage estimation because no matched parental heterozygous pair was available for amplification-bias correction. These blood-based results do not support UPD(14)mat/Temple syndrome at the assayed loci, but tissue-restricted UPD(14) or imprinting mosaicism cannot be completely excluded. The most supportable interpretation is therefore mosaic trisomy 14 supported by cytogenetic and SNP-CMA findings, with 14q32.2 methylated-allele dosage reflecting the broader chromosome 14 gain and quantitative STR data supporting excess paternal dosage rather than a separately demonstrated imprinting abnormality.

### 3.5. Limitations

Several limitations should be emphasized. First, the quantitative discordance between 6/100 abnormal cultured metaphases and approximately 48–50% mosaic estimates from two DNA-based assays remains unresolved; similar DNA-based estimates do not resolve this discrepancy, and no independent quantitative assay on uncultured cells or non-blood tissue was available. Second, the array does not directly interrogate the repetitive short arm of acrocentric chromosome 14; it demonstrates a near chromosome-wide 14q gain compatible with the cytogenetic trisomy 14 finding. Third, the targeted 14q32.2 assay provided absolute methylated and unmethylated copy numbers, but the clinical report did not provide validated proportional methylation reference intervals; therefore, no independent epimutation is inferred. Fourth, STR and methylation analyses were performed in peripheral blood only, and the parental dosage origin of the third chromosome 14 copy was not established. Fifth, the combined insulin–clonidine stimulation test was incompletely documented and is insufficient to establish GHD. Sixth, the formal rhGH indication was SGA with persistent short stature, but serial early-childhood measurements were insufficient to reconstruct the timing of failed spontaneous catch-up growth. Finally, three months is too short to assess treatment efficacy, scoliosis risk, metabolic safety, pubertal effects, or adult-height outcome; the interval findings are descriptive only.

## 4. Conclusions

**Figure 4:**
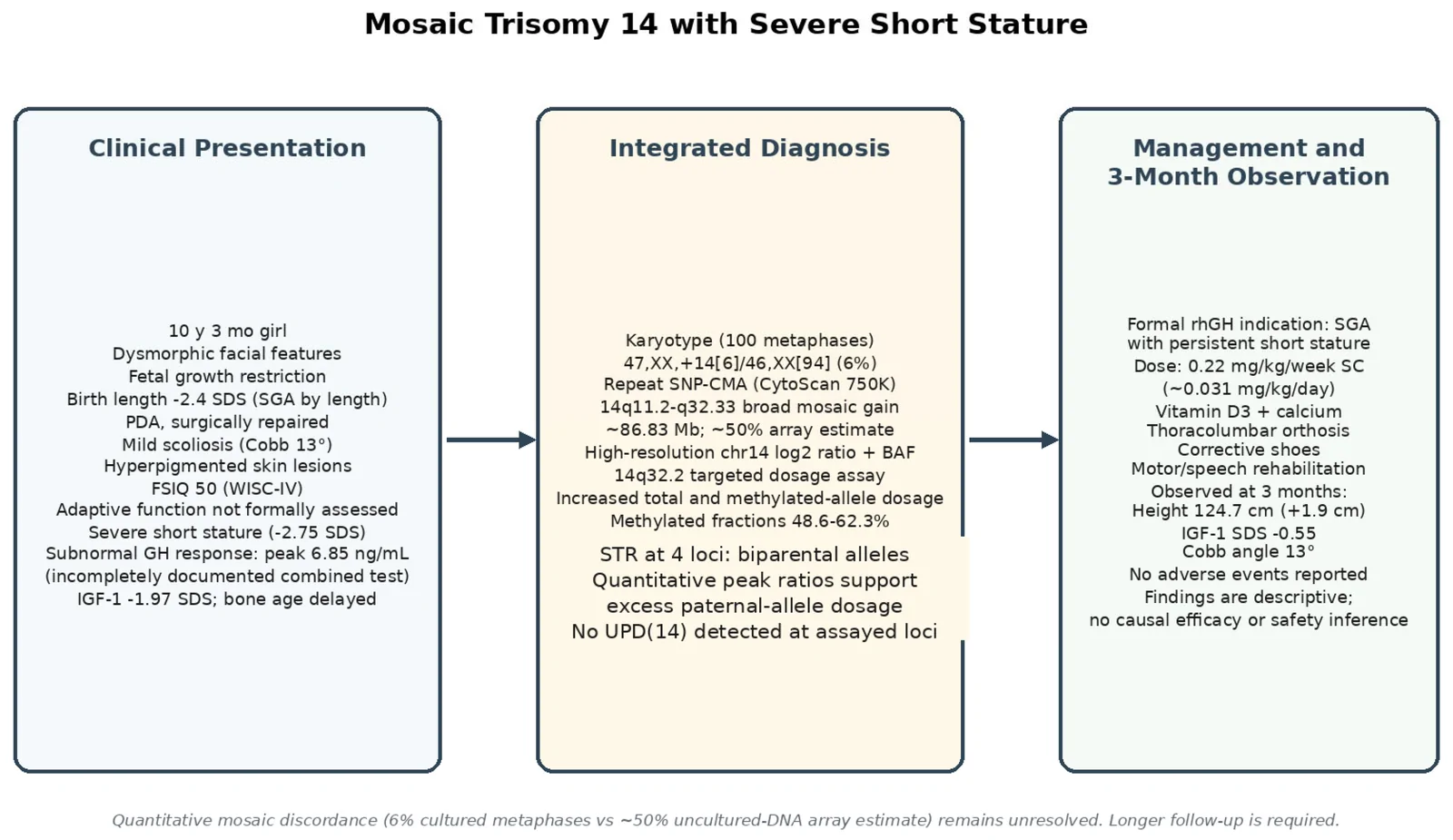
Graphical abstract summarizing the clinical presentation, integrated diagnosis, treatment indication, and three-month observations. The patient had mosaic trisomy 14, severe persistent short stature after SGA birth by length, and a subnormal GH response on an incompletely documented combined stimulation test that was insufficient to establish GHD. Karyotyping showed 47,XX,+14[6]/46,XX[94], and repeat SNP-based CMA demonstrated a broad 14q11.2-q32.33 mosaic gain with an array-estimated fraction of approximately 50%; the quantitative discrepancy between cultured metaphases and DNA-based estimates remains unresolved. The 14q32.2 assay showed increased total and methylated-allele dosage with methylated fractions of 48.6–62.3%. Four STR loci showed biparental inheritance, and quantitative peak-height/peak-area analysis at three informative markers supported excess paternal-allele dosage and therefore a paternal origin of the additional chromosome 14, although the assignment remains supportive rather than definitive. rhGH was prescribed for SGA with persistent short stature at 0.22 mg/kg/week. The three-month height, IGF-1, and Cobb-angle findings are reported descriptively, and no causal efficacy or safety inference is made.

Mosaic trisomy 14 is a rare disorder with multisystem involvement and variable tissue distribution. In this case, G-banded karyotyping identified trisomy 14 in 6 of 100 cultured peripheral-blood metaphases, while repeat SNP-based CMA demonstrated a near chromosome-wide 14q11.2-q32.33 mosaic gain with an array-estimated mosaic fraction of approximately 50%, compatible with the cytogenetic diagnosis; the quantitative difference between cultured metaphases and uncultured-DNA estimates remains unresolved. Targeted 14q32.2 analysis showed increased total and methylated-allele dosage, with methylated fractions of 48.6–62.3%, without establishing an independent epimutation. Four STR loci showed biparental inheritance and no evidence of UPD(14) at the assayed loci in peripheral blood; quantitative peak-height and peak-area ratios at three informative markers demonstrated excess paternal-allele dosage and provided supportive, but not definitive, evidence for paternal origin of the additional chromosome 14. The combined insulin–clonidine study yielded a subnormal GH response but was insufficiently documented to establish GHD. rhGH was prescribed for SGA with persistent severe short stature at 0.22 mg/kg/week. The three-month changes in height, IGF-1, and Cobb angle are descriptive observations rather than evidence of efficacy or safety. Integrated cytogenetic and molecular evaluation, cautious interpretation of imprinting assays, and longitudinal multidisciplinary follow-up remain important in chromosome 14 mosaicism. The clinical presentation, diagnostic pathway, and management are summarized in Figure 4.

## Figures and Tables

**Table 1:** Combined insulin–clonidine GH stimulation test results.

Time (min)	0	15	30	60	90	120
GH (ng/mL)	0.75	-	1.99	5.65	6.85 *	-

* Peak GH was 6.85 ng/mL at 90 min. Contemporaneous medication orders documented insulin 0.075 U/kg and clonidine 0.096 mg orally (approximately 4 µg/kg). The archived laboratory report provided GH values at 0, 30, 60, and 90 min; the 15 and 120 min GH fields were blank. Serial glucose concentrations, exact administration times, and the original assay/calibration details were not available, so the combined session is incompletely interpretable and is not considered sufficient to establish GHD. The result is reported descriptively as a subnormal stimulated GH response.

**Table 2:** Methylation-specific copy-number and STR linkage results at the 14q32.2 imprinting region.

Target	Chromosomal Position (GRCh38/hg38)	Total Copy Number	Methylated-Allele Copy	Unmethylated Allele Copy	Methylated Fraction/Interpretation
IG-DMR-A	chr14:100,810,552-100,810,727	2.57	1.42	1.15	55.3%; increased methylated-allele dosage
IG-DMR-B	chr14:100,811,123-100,811,291	2.52	1.57	0.95	62.3%; increased methylated-allele dosage
MEG3-DMR-A	chr14:100,825,816-100,825,919	3.13	1.58	1.55	50.5%; increased methylated-allele dosage
MEG3-DMR-B	chr14:100,825,962-100,826,045	2.63	1.57	1.06	59.7%; increased methylated-allele dosage
MEG3-DMR-C	chr14:100,826,190-100,826,289	2.69	1.62	1.07	60.2%; increased methylated-allele dosage
MEG8-DMR-A	chr14:100,956,746-100,956,901	2.66	1.30	1.36	48.9%; increased methylated-allele dosage
MEG8-DMR-B	chr14:100,982,881-100,983,015	2.49	1.21	1.28	48.6%; increased methylated-allele dosage
Targeted 14q32.2 copy-number interval	chr14:100,810,639-100,982,948	172.3 kb	-	-	Within broader 14q gain; independent duplication not established
STR G14S19 (Father/Mother)	-	Patient 368/385	Father 368/385	Mother 385/385	Biparental; paternal dosage supported *
STR G14S23 (Father/Mother)	-	Patient 194/198	Father 198/200	Mother 194/198	Biparental; paternal dosage supported *
STR G14S26 (Father/Mother)	-	Patient 430/432	Father 430/438	Mother 430/432	Biparental; paternal dosage supported *
STR G14S28 (Father/Mother)	-	Patient 456/462	Father 450/462	Mother 452/456	Biparental; parent-specific alleles; not ratio-normalized *

* Allele-dosage interpretations derived from STR fragment analysis, marked with an asterisk in the table, are reported descriptively and are not considered definitive proof of parent of origin.

**Table 3:** Clinical parameters before treatment and at 3 months of treatment.

Parameter	Before Treatment (26 January 2026)	After 3 Months of Treatment (26 April 2026)
Height (cm)	122.8	124.7
Height SDS (Chinese age- and sex-specific growth reference)	−2.75	−2.55
Weight (kg)	24.0	25.1
BMI (kg/m^2^)	15.92	16.13
Scoliosis	Right-convex thoracolumbar, Cobb 13°	Right-convex thoracolumbar, Cobb 13°
Bone age (Greulich–Pyle)	6 years 8 months (delay ~3 y 7 mo)	6 years 11 months
IGF-1 (µg/L)	87	145
IGF-1 SDS (age- and sex-adjusted)	−1.97	−0.55
25(OH)D (µg/L)	21.47	32
Fasting glucose (mmol/L)	4.56	4.83
HbA1c (%)	5.2	5.2
Liver, kidney, thyroid function	Normal	Normal
Adverse events	N/A	No adverse events reported during 3 months
Adherence	N/A	>95%

**Table 4:** Auxological, endocrine, and imaging timeline.

Date/Age	Height (cm)/SDS	Weight (kg)	Investigations and Notes
Birth (37 weeks)	43 cm, −2.4 SDS	2.4 (−1.1 SDS)	FGR, oligohydramnios; vaginal delivery; no asphyxia
5 months	-	-	Head control and tooth eruption (delayed milestone)
8 months	-	-	Independent sitting (delayed milestone)
~1 year	-	-	Continuous murmur; PDA diagnosed by echocardiography
1.5 years (18 months)	-	-	Surgical PDA closure; first words (delayed milestone)
20 months	-	-	Independent walking (delayed milestone)
26 January 2026 (10 y 3 mo)	122.8 cm, −2.75 SDS	24.0	Admission; GH peak 6.85 ng/mL; IGF-1 87 µg/L (−1.97 SDS); 25(OH)D 21.47 µg/L; bone age 6 y 8 mo; Cobb 13°; WISC-IV FSIQ 50; karyotype 47,XX,+14[6]/46,XX[94]
27 January 2026	-	-	rhGH initiation for SGA with persistent short stature (0.22 mg/kg/week SC)
26 April 2026 (3 months)	124.7 cm, −2.55 SDS	25.1	IGF-1 145 µg/L (−0.55 SDS); 25(OH)D 32 µg/L; Cobb 13°; HbA1c 5.2%; no adverse events reported during 3 months

SDS values were derived from INTERGROWTH-21st newborn standards for birth measurements and from age- and sex-adjusted Chinese growth reference curves for current measurements; IGF-1 SDS was calculated using the Siemens Atellica IGF-1 analyzer normative reference. Bone age was assessed using the Greulich–Pyle method.

**Table 5:** Comparison of clinical features of mosaic trisomy 14, UPD(14)mat/Temple syndrome, and UPD(14)pat/Kagami–Ogata syndrome, with the present case.

Feature	Mosaic Trisomy 14 [4,16,18]	UPD(14)mat/Temple Syndrome [6]	UPD(14)pat/Kagami–Ogata Syndrome [7,10,11]	Present Case
IUGR/FGR	Reported in published cases	Reported	Reported	Yes
Postnatal growth failure	Reported in published cases	Reported	Reported	Yes (−2.75 SDS)
Cognitive/adaptive function	Developmental or cognitive impairment reported; heterogeneous	Developmental delay may occur	Developmental impairment may occur	FSIQ 50; adaptive functioning not formally assessed
Dysmorphic facial features	Reported	Reported but variable	Characteristic facial phenotype reported	Yes (hypertelorism, low nasal bridge)
Congenital heart defect	Reported, including PDA/VSD	Reported but not defining	Reported but not defining	PDA, repaired
Scoliosis/skeletal findings	Skeletal abnormalities reported	Variable skeletal findings	Thoracic and skeletal abnormalities are characteristic	Mild scoliosis (Cobb 13°)
Abdominal-wall defects	Not a defining feature	Not a defining feature	Characteristic in many cases	None
Polyhydramnios	Not a defining feature	Not characteristic	Characteristic prenatal feature	No; oligohydramnios
Joint contractures	Not a defining feature	Not characteristic	Characteristic feature	None
Feeding difficulty	Reported in some cases	Reported	Reported, especially neonatally	No
Pigmentary skin lesions	Reported in mosaic trisomy 14	Not defining	Not defining	Hyperpigmented macules
Precocious puberty	Not established as characteristic	Reported/characteristic tendency	Not typical	Prepubertal at current assessment
14q32.2 methylation/dosage	Depends on parental dosage and associated imprinting state	Loss-of-methylation pattern	Paternalization/gain-of-methylation pattern	Methylated fractions 48.6–62.3% with increased total copy number; interpreted as dosage effect, not independent epimutation
STR/parental inheritance	UPD may coexist after trisomy rescue	Maternal-only in UPD(14)mat	Paternal-only in UPD(14)pat	Biparental alleles at 4 loci; quantitative peak-height/area ratios at 3 bias-correctable informative loci support excess paternal-allele dosage; paternal origin supported but not definitive
GH stimulation/GHD	GHD not established as a characteristic feature	GHD and rhGH treatment have been reported [22]	GHD not characteristic	Subnormal GH response (peak 6.85 ng/mL) in incompletely documented combined test; GHD not established

FSIQ: full-scale intelligence quotient; IUGR/FGR: intrauterine growth restriction/fetal growth restriction; PDA: patent ductus arteriosus; VSD: ventricular septal defect. Categorical descriptors were minimized because published mosaic trisomy 14 series are small and heterogeneous.

## Data Availability

The data supporting the findings of this case report are contained within the article, tables, and figures. Raw laboratory reports are archived at the institutions of origin and may be available from the corresponding author upon reasonable request, subject to institutional requirements and the consent of the patient’s legal guardians.

## Supplementary Materials

The following supporting information can be downloaded at: https://www.mdpi.com/article/10.3390/genes17091123/s1.

## Abbreviations

BMI	Body mass index
CMA	Chromosomal microarray analysis
CNV-seq	Copy-number variation sequencing
DMR	Differentially methylated region
DXA	Dual-energy X-ray absorptiometry
ECG	Electrocardiogram
FISH	Fluorescence in situ hybridization
FSH	Follicle-stimulating hormone
GH	Growth hormone
GHD	Growth hormone deficiency
HbA1c	Glycated hemoglobin
IGF-1	Insulin-like growth factor-1
IGFBP-3	Insulin-like growth factor binding protein-3
IQ	Intelligence quotient
ISCN	International System for Human Cytogenomic Nomenclature
KOS	Kagami–Ogata syndrome
LH	Luteinizing hormone
MRI	Magnetic resonance imaging
NIPT	Non-invasive prenatal testing
PDA	Patent ductus arteriosus
rhGH	Recombinant human growth hormone
SDS	Standard deviation score
SGA	Small for gestational age
SNP	Single-nucleotide polymorphism
STR	Short tandem repeat
TI-RADS	Thyroid Imaging Reporting and Data System
TS14	Temple syndrome
UPD	Uniparental disomy
UPD(14)mat	Maternal uniparental disomy of chromosome 14
UPD(14)pat	Paternal uniparental disomy of chromosome 14
25(OH)D	25-hydroxyvitamin D
