# Supplementary material for: Mosaic Trisomy 14 with Severe Short Stature: A Case Report
Source: Genes (Basel). 2026 Sep 15;17(9):1123. doi: 10.3390/genes17091123 (PMC13606590; doi:10.3390/genes17091123)
Supplement: Supplementary file 1 [file genes-17-01123-s001.zip › Supplementary_Materials_new.pdf]

Supplementary Materials

Supplementary Figure S1. Quantitative STR electropherograms at informative 14q32.2 loci.

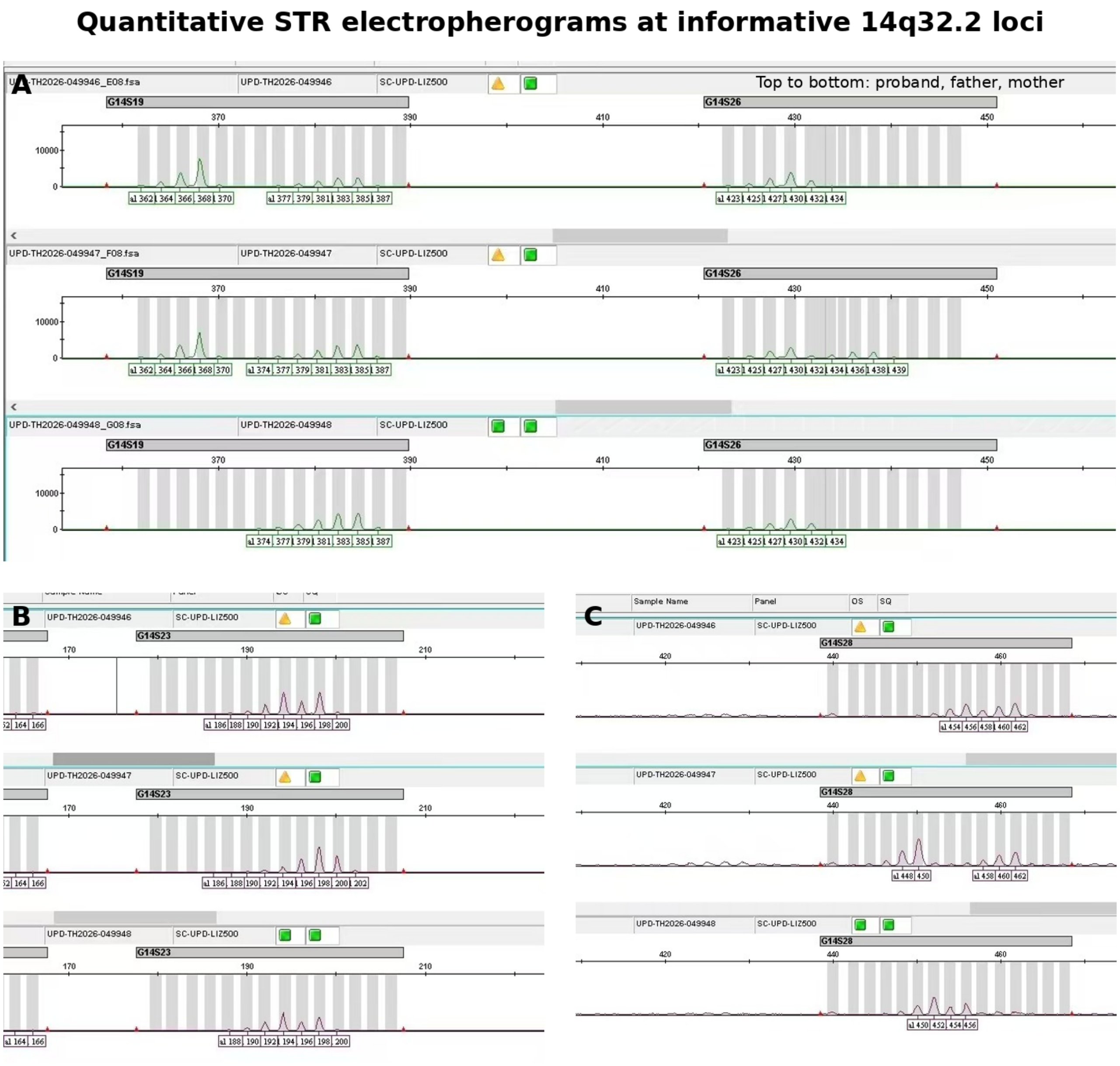

A: G14S19 and G14S26; B: G14S23; C: G14S28. In each electropherogram, traces are ordered proband, father, mother from top to bottom.

Panel A shows G14S19 and G14S26, Panel B shows G14S23, and Panel C shows G14S28. For each locus, traces are ordered proband, father, and mother from top to bottom. The electropherograms demonstrate biparental inheritance. G14S19, G14S23, and G14S26 were used for quantitative dosage analysis after correction for allele-specific PCR amplification bias using a parent with the same heterozygous allele pair. G14S28 distinguishes the paternal-specific 462-bp allele from the maternal-specific 456-bp allele but was not included in the normalized quantitative mean because no matched parental heterozygous pair was available for amplification-bias correction.

Supplementary Table S1. Quantitative 14q32.2 STR dosage analysis.

Marker	Proband genotype	Father genotype	Mother genotype	Paternal allele	Maternal allele	Normalized peak-height ratio	Normalized peak-area ratio
G14S19	368/385	368/385	385/385	368	385	1.63	1.62
G14S23	194/198	198/200	194/198	198	194	1.36	1.39
G14S26	430/432	430/438	430/432	430	432	1.26	1.28
G14S28	456/462	450/462	452/456	462	456	Not used for normalized mean	Not used for normalized mean

Notes: For G14S19, the father shared the same 368/385 heterozygous allele pair as the proband and was used to correct allele-specific amplification bias. For G14S23 and G14S26, the mother shared the same heterozygous allele pair as the proband and was used for bias correction. The normalized paternal:maternal ratio was calculated by dividing the proband paternal:maternal peak ratio by the corresponding same-pair parental peak ratio. Mean normalized ratios across the three bias-correctable loci were 1.42 by peak height and 1.43 by peak area. In a theoretical 50% trisomic mixture containing one additional paternal chromosome 14 copy, the expected paternal:maternal dosage ratio approaches 1.5. The observed values therefore support excess paternal-allele dosage but are not considered definitive proof of parent of origin. The underlying electropherograms and quantitative peak metrics were obtained from the archived clinical STR fragment-analysis files.
